# Exploring the relationships between dominance behavioral system, mentalization, theory of mind and assertiveness: analysis in a non-clinical sample

**DOI:** 10.3389/fpsyg.2024.1407933

**Published:** 2024-07-15

**Authors:** Francesco Cerciello, Clara Esposito, Ilaria La Penna, Luigia Simona Sica, Alessandro Frolli

**Affiliations:** ^1^Disability Research Centre, Department of International Humanities and Social Sciences, Rome University of International Studies, Rome, Italy; ^2^Child Neuropsychiatry Outpatient Clinic, Fondazione Italiana Neuroscienze e Disordini dello Sviluppo - FINDS, Caserta, Italy; ^3^Department of Humanities, University of Naples Federico II, Naples, Italy

**Keywords:** dominance behavioral system, mentalization, theory of mind, assertiveness, motivational system

## Abstract

**Introduction:**

The dominance behavioral system, a fundamental aspect of human behavior, orchestrates the drive for dominance, regulates dominant-subordinate dynamics, and shapes responses to perceived power dynamics. While the existing literature extensively delves into the components of this system, scant attention is paid to its interplay with mentalization, theory of mind, and assertiveness. Moreover, gender disparities in dominance behaviors are largely studied in terms of biological variables (levels of testosterone) and clinical populations. This study aims to understand the relationships between activation strategies of the dominance behavioral system, mentalization processes, theory of mind abilities, and levels of social discomfort in assertive communication. Moreover, to identify gender differences in the dominance behavioral system in a non-clinical sample.

**Methods:**

Our sample was composed of 67 students from a non-clinical population. They claimed the absence of any psychological, neurological, or developmental disorders.

**Results:**

A regression analysis was performed, and we found that levels of mentalization predict levels of hyperactivation of dominance behavioral system, but no significant results for the deactivation levels of the system were found. Moreover, no gender differences were found in levels of activations of the dominance behavioral system.

**Conclusion:**

These findings underscore the pivotal role of mentalization abilities in interpersonal dynamics, emphasizing the need for individuals to navigate social interactions adeptly. Furthermore, our research unveils implications for individual well-being and psychopathology, urging further investigation into how these dimensions intersect with various psychological disorders. By discerning the intricate mechanisms at play, we can develop targeted therapeutic interventions tailored to specific behavioral patterns, ultimately enhancing psychological resilience and fostering healthier social relationships in a non-clinical population.

## 1 Introduction

### 1.1 The behavioral motivational systems: the dominance behavioral system

Behavioral motivational systems are sets of reactions that are triggered under circumstances of individual engagement with the societal context, directed towards ensuring survival. Behavioral motivational systems are set into motion in reaction to stimuli pertinent to their objective and cease functioning upon goal achievement or when the impossibility of achievement arises (Mikulincer and Shaver, 2012). The cognitive-evolutionary theory of interpersonal motivational systems is based on a broader understanding of motivation in behavior regulation systems, both towards the impersonal environment and the social one. Some of these behavioral regulation systems are intrinsically innate, meaning they are based on dispositions or action tendencies selected through evolutionary processes (Tombolini and Liotti, 2005). Various behavior regulation systems emerge, known as “motivational systems” because they guide behaviors and mental states towards desirable goals (Liotti, 2001a, 2005). Other authors, such as Cosmides (1989), define motivational systems as algorithms for processing environmental information concerning bodily information and define interpersonal motivational systems as algorithms for processing social information (Cosmides, 1989). Additionally, these systems regulate conduct based on specific goals and are closely linked to emotional experience. Emotions accompany the action of these systems and can be considered indicators of their activity. According to Liotti (2001b), each emotional experience can be better understood when related to the motivational system it belongs to. It is possible to identify five distinct interpersonal motivational systems based on the different goals that they aim to achieve: the attachment system, the caregiving system, the sexual system, the competitive (or agonistic) system, and the cooperative system. It is important to clarify that the competitive (or agonistic) system and the behavioral system of dominance are terms used interchangeably here. All these systems converge towards an innate goal, which represents a fundamental “value” for survival and environmental adaptation (Edelman, 1992). Each interpersonal motivational system is characterized not only by a specific goal but also by a precise activator (Liotti, 2001b). An example is the attachment system, which is activated by sensations of fatigue, physical pain, emotional discomfort, and loneliness; its goal is to obtain the protective proximity of an available figure who can offer help, comfort, and protection. The caregiving system is activated by signals indicating the need for protection; its purpose is to provide protection and comfort to those perceived as more vulnerable and in difficulty. The sexual system is activated by signals from potential sexual partners and aims at mating. The cooperative system is activated in the presence of resources that are not perceived as limited but accessible to all individuals involved, and by signals indicating the absence of agonistic threats, such as a smile; its objective is to pursue a common goal through collaboration. Finally, the dominance behavioral system (DBS) is the focus of the present research study: it is activated in the presence of resources perceived as limited, signals of challenge from a peer, and, in humans, also by ridicule, guilt, and judgment; its goal is to establish hierarchies of dominance and submission (Tombolini and Liotti, 2005). This system can be conceptualized as an innate system that governs the urge for dominance, dominant-subordinate behavior, and responsiveness to perceptions of power and subservience (Johnson et al., 2012). The dominance behavioral system manages interpersonal transactions grounded in competition and strives to attain material and social authority over resources to enhance power and survival prospects (Shaver et al., 2011). Johnson et al. (2012) introduce three distinct terms to describe the components of the DBS: dominance motivation, dominance behavior, and power, which encompasses self-perceptions related to these aspects. Dominance motivation characterizes an individual’s desire and vigor in seeking power, a concept that aligns closely with Winter’s (1994) conception of the power motive. Dominance motivation also influences individuals’ perceptions of their life goals. Individuals with high dominance motivation are more inclined to seek the admiration and social attention of others. Consequently, dominance motivation is linked to the establishment of life goals that prioritize external validation, such as aspirations for fame and wealth (Duriez et al., 2007). Dominance behaviors are commonly characterized by observing the results of aggressive interactions among conspecifics. In studies involving nonhuman primates, for instance, dominance behavior is often delineated within a dyadic interaction framework. In such interactions, one member of the dyad exhibits aggressive behavior or displays gestures indicative of aggression, while the other responds with submissive behaviors (Bernstein, 1980). In human research, definitions of dominance behavior tend to encompass broader criteria compared to those in ethology. Firstly, researchers in psychology often emphasize behaviors aimed at increasing power, regardless of their effectiveness in achieving this objective (Anderson and Kilduff, 2009). Secondly, dominance behaviors in humans extend beyond competitive actions; they also encompass behaviors aimed at currying favor with authorities, forming coalitions, and assertiveness (Mazur and Booth, 1998). Power has been defined as “the ability to provide or withhold valued resources or administer punishments” to others (Anderson and Berdahl, 2002, p. 1362). Resources may be physical or social, such as higher esteem from others, praise, and positive attention (Hawley, 1999). Each behavioral motivational system possesses a primary strategy that embodies the most effective behavioral model for accomplishing the objective. Nevertheless, if an individual’s life experiences expose the primary strategy as ineffective, it is substituted with dysfunctional alternate strategies: specifically, hyperactivation and deactivation strategies (Mikulincer and Shaver, 2012). Hyperactivation strategies amplify the utilization of the primary strategy, leading to persistent activation of the system and engendering a state of agitation and tension. Deactivation strategies diminish the utilization of the primary strategy, and the system struggles to activate, resulting in a constriction of personal experience and a decline in social engagement (Mikulincer and Shaver, 2012). Stimuli activating this system encompass restricted resource access, indications of challenge and threat to one’s authority, and situations involving evaluation or judgment (Shaver et al., 2011). Conversely, stimuli deactivating the system encompass signals of deference from others, indicating their inferiority, or signals of superiority from others, confirming their dominance (Shaver et al., 2011). The primary strategy of the dominance behavioral system entails behaviors directed at acquiring or sustaining a dominant position, asserting authority, rights, and proficiency. Hyperactivation strategies involve aggressive, hostile, and irate behaviors towards perceived rivals, with a propensity to confront even in the presence of minimal or ambiguous threat cues (Shaver et al., 2011). Deactivation strategies entail relinquishing the struggle to defend against threats; submissive behaviors are triggered, and efforts to acquire power are forsaken even in the face of explicit aggressions or provocations (Shaver et al., 2011). Submissive behaviors manifest as heightened discomfort and social reticence in potential conflict scenarios (Gilbert and Allan, 1994). Dominant behaviors reflect a strong inclination of the individual to seize power and manifest as confrontational interactions with peers (Johnson et al., 2012).

### 1.2 DBS and theory of mind, mentalization and assertiveness

Each signal capable of activating a system corresponds to a related emotional experience. Emotions thus have considerable cognitive value regarding the interpersonal motivational setup: they indicate towards which interpersonal goal the “impulse to act” is directed (Frijda, 1990), perceived in oneself or others, and whether an obstacle is being encountered in pursuing it. The schemas related to self-knowledge and knowledge of the other within a relationship, defined by Safran and Segal (1993) as “interpersonal schemas” and by Bowlby (1969, 1973) as “internal working models,” are subject to the dynamics of assimilation and accommodation (Flavell, 1963). Emotions, therefore, are modes of functioning of the motivational systems and can be perceived at a conscious level. When two people meet, their intersubjective exchange is always regulated and motivated by these systems, which activate accordingly. Additionally, these systems are physiological regulation mechanisms that, once activated, organize social and interpersonal behavior, influencing emotional experience and the representation of the “self-with-the-other” (Liotti, 2001b). Consequently, every human emotion presupposes the intervention of higher cognitive processes and therefore also involves complex cognitive processes that modulate and give meaning to them within social interactions, such as social cognition, i.e., the cognitive processes through which one’s interactions with others are understood, processed, and remembered (Morgan et al., 2017). The term social cognition generally refers to the mental operations underlying social interactions, including perceiving, interpreting, and generating responses to the intentions, dispositions, and behaviors of others (Green et al., 2008). The recognition that individuals’ actions can be predicted and comprehended through mental states such as beliefs, desires, emotions, and intentions is termed Theory of Mind (ToM) (Wellman, 2014). Specifically, ToM can be divided into “affective” and “cognitive” components. This denotes that an individual can attribute and/or grasp intentions, thoughts (cognitive ToM), or feelings (affective ToM). It is a comprehensive construct that relates to the capacity to deduce thoughts and emotions, to contemplate the mental states of others, and to the interpersonal connections that each of us maintains with other human beings (Westby, 2014). In social contexts, where interpersonal interactions are based on perceived levels of authority, those who perceive high levels of authority tend to experience positive emotions, while those who perceive low levels of authority tend to experience negative emotions (Keltner et al., 2003; Cho and Keltner, 2020). Moreover, individuals with high levels of authority tend to focus their attention on social situations with a high potential for reward, viewing other individuals as instruments for achieving their objectives. Conversely, individuals with low levels of authority tend to direct their attention toward potential threats, perceiving themselves as instruments for achieving the objectives of others (Keltner et al., 2003; Cho and Keltner, 2020). Authority not only affects an individual’s emotions but also impacts their interpersonal connections with colleagues in the workplace, neighbors, friends, family members, and romantic partners (Keltner et al., 2003; Anderson et al., 2012). A higher-order level of social cognition is represented by the ability to understand and reason about one’s own and others’ mental and affective states, using this understanding to solve problems and manage subjective suffering, a level defined as mentalization (Bateman and Fonagy, 2013; Luyten and Fonagy, 2014). In the domain of interpersonal relationships, Fonagy (1989) introduced the concept of “mentalization.” This term (used interchangeably here with the term “reflective functioning”) was initially popularized through the study of Borderline Personality Disorder (BPD) (Fonagy, 1989; Fonagy, 1991; Fonagy and Bateman, 2008; Fonagy and Luyten, 2009) and parent–child attachment (Fonagy et al., 1991, 2007). The concept of mentalization pertains to the ability to reflect on internal mental states such as feelings, desires, goals, and attitudes, both regarding oneself and others. Research indicates that this ability develops within the context of secure attachment relationships. Conversely, disruptions in attachment relationships, likely in interaction with environmental and genetic vulnerabilities, have been associated with deficits in mentalization. Such deficits have been demonstrated to play a pivotal role in a range of disorders and problematic behaviors such as BPD (Bateman and Fonagy, 2004), eating disorders (Skårderud, 2007b), depression (Luyten et al., 2012), and antisocial personality disorder (Bateman and Fonagy, 2008). Two broad types of compromises in mentalization have been described and shown to be implicated as vulnerability factors for the development of psychopathology (Fonagy et al., 2002; Fonagy and Luyten, 2016). The first compromise concerns what is termed hypo-mentalization, indicating an incapacity to regard one’s mind and/or that of others as complex models. Hypo-mentalization has been associated with susceptibility to a wide range of disorders, including BPD (Fonagy and Luyten, 2016), eating disorders (Skårderud, 2007b), and depression (Lemma et al., 2011; Luyten and Fonagy, 2014). Individuals exhibiting the opposite inclination, hyper-mentalization, also known as excessive mentalization (Sharp et al., 2011), may introduce a different form of distortion into their self-reports. Hyper-mentalization entails generating mental representations of actions without adequate evidence to support these representations. The tendency to develop inaccurate models of one’s mind and others’ minds is typical of individuals who provide lengthy and excessively detailed accounts that bear little or no relationship with observable (testable) reality. Additionally, individuals prone to hyper-mentalization may perceive themselves as particularly adept at mentalizing and, consequently, may also display distorted (excessive) responses to self-report measures (Fonagy et al., 2016). This capacity to comprehend one’s inner mental states of beliefs, desires, needs, and those of others can also pertain to drawing conclusions based on external cues (e.g., facial expressions and gestures). However, it can also relate to understanding someone’s internal experience based on what one knows about the individual and the situation they find themselves in (internal versus external mentalizing) (Lüdemann et al., 2021). Consistent with various authors (Mikulincer and Shaver, 2012; Salzano et al., 2023), hyperactivation and deactivation strategies of the agonistic system correspond to aggressive behaviors towards rivals and feelings of reluctance in assertively communicating one’s own needs and/or emotions. The ability to communicate effectively is defined as “assertiveness” (Ahmadi et al., 2014). Assertiveness is one of the most important social skills (Motahari et al., 2019) that contributing to sociocultural adaptation (Lee and Ciftci, 2014). It has been defined as the ability to express emotions, beliefs, and thoughts explicitly. Furthermore, it might be considered as the ability to defend one’s ideals truthfully (Ahmadi et al., 2014), enabling the individual to act to assert their rights without violating those of others (Speed et al., 2018). Individuals use assertiveness in social interactions also to create a sense of desirability (Baker and Jeske, 2015). Indeed, this could lead to increased self-esteem (Motahari et al., 2019), social support (Rahimian Boogar et al., 2007), quality of interpersonal interactions (Lin et al., 2004; Manesh et al., 2015), improvement in psychological status (Mc Cabe and Timmins, 2003), and reduction in social anxiety (Lin et al., 2004; Manesh et al., 2015). One of the most important factors in a healthy interpersonal relationship is the ability to be assertive (Motahari et al., 2019), which can lead to effective relationships with others (Tavangar et al., 2014). Individuals who fail to be assertive experience many problems including depression, disappointment, anger, anxiety, poor social communication, physical complaints, and family problems (Samouei et al., 2014). The results of Khazaie et al. (2014) showed that those who lack assertive behaviors have lower self-esteem and higher social anxiety, associated with high shyness and aggression (Khazaie et al., 2014). People who fail to positively interact with others due to a lack of assertiveness or interpersonal effectiveness will experience great anxiety when interacting with a group, and this may disrupt their social and occupational functioning (Tavangar et al., 2014).

### 1.3 Gender differences

In men, high levels of testosterone appear to encourage behaviors aimed at domination, that is, improving one’s status relative to that of others (Mazur and Booth, 1998). Generally, dominant behavior seems aggressive, with the apparent intent to inflict harm on another person, but often dominance can also be expressed in a non-aggressive manner (Mazur and Booth, 1998). This observation is supported by various studies highlighting how testosterone influences human behavior (Giammanco et al., 2005). Testosterone is an androgenic and anabolic sex hormone, which is crucial for the development of primary and secondary male characteristics (Taulbjerg et al., 2021). It has complex effects on psychological traits and behavior; it is associated with social dominance and competition and is a potential human sex pheromone (Liu et al., 2024). Prevailing theories suggest that testosterone should directly boost competitive and dominant behaviors during periods of social competition or challenge (Mazur and Booth, 1998; Archer, 2006). Consistent with this challenge hypothesis is evidence that higher testosterone is positively related to aggressive and dominant behaviors across a variety of non-human animal species, especially during times of social instability (Muller and Wrangham, 2004; Archer, 2006; the biosocial model of status makes similar predictions, Mazur and Booth, 1998; Terburg and van Honk, 2013). Support for the challenge hypothesis has also emerged in human studies as well. Indeed, a compelling line of research demonstrates that testosterone administration enhances neural, attentional, and behavioral responses to social signals of dominance threat (Hermans et al., 2008; Bos et al., 2012; Terburg et al., 2012; Terburg and van Honk, 2013; Enter et al., 2014; Goetz et al., 2014; Radke et al., 2015). It is important to note that while testosterone can influence behavior, it’s not the sole determining factor. Environment, education, life experiences, and other biological and psychological factors all play a role in shaping an individual’s behavior. Consequently, dominant behavior can vary widely among individuals and in different contexts (Mehta et al., 2015). Hence, the idea arose to test whether gender could be somehow linked to the dominance behavioral system in our sample.

### 1.4 Aims and objectives of the study

The activation of interpersonal motivational systems, as well as relational schemas, can influence intersubjectivity and the mental capacities necessary to manage life tasks and interpersonal relationships. This is why we considered exploring how mentalization and theory of mind are connected to the hyperactivation and deactivation of behavioral systems, specifically the dominance motivational system. In conclusion, the theoretical assumption of the present study is that mentalization abilities, theory of mind skills, and an assertive communication style may be closely linked to hyperactivation and deactivation strategies of the dominance behavioral system. The main aim is to understand the relationships between activation strategies of the dominance behavioral system, mentalization processes, theory of mind abilities, and levels of social discomfort in assertive communication. Moreover, the study aims to identify gender differences in levels of deactivation and hyperactivation of the dominance behavioral system: we expect that males are more activated than females. Finally, the research hypothesis to be tested is that levels of hyperactivation and deactivation in a non-clinical population are predicted by scores of hyper- and/or hypo-mentalization, performance on theory of mind tests, and levels of social discomfort in assertive communication.

## 2 Materials and methods

### 2.1 Participants

Students from different areas of Rome (Italy) were recruited for the study. To be included in the study, participants had to declare: (a) absence of current or previous psychiatric or psychological diagnosis; (b) absence of neurological, neuropsychological or neurodevelopmental disorders; (c) Italian as mother tongue; (d) aged between 18 and 31 years. The total sample was 81 participants, but 14 were excluded as they did not meet the inclusion criteria, specifically as they reported the presence of psychopathologies. Lastly, 67 individuals were included in the study (27 males and 40 females; age range: 18–30 years, mean = 25.71 years, SD = 2.61). Participants were asked to report their biological sex. There were no participants who identified themselves in a gender other than their biological gender (e.g., transgender, non-binary etc.). The research was conducted after participants had signed informed consent and in accordance with the Ethical Standards of the Declaration of Helsinki and the approval (code: S.5–0923) of the Ethics Committee of Department of International Humanities and Social Sciences, Rome University of International Studies (UNINT). Details about the sociodemographic characteristics of the participants are available in Table 1. Data were collected and analyzed by the Disability Research Centre (DRC) at the Department of International Humanities and Social Sciences, Rome University of International Studies.

**Table 1:** Descriptive statistics of the sample.

	**Statistics**
Number	67
Males/Females (*n*)	27/40
Age (y)	25.71 (2.61)
Education (*n*)	Middle school graduation: 1 High school graduation: 15 Bachelor’s degree: 24 Master’s degree 27

### 2.2 Measures

The Power Behavioral System Scale (PBSS, Shaver et al., 2011) is a self-assessment questionnaire consisting of 28 items that measures a person’s overall orientation towards power and assertion. The scale assesses the two main secondary power strategies: deactivation (De) and hyperactivation (Hy). The 14 deactivation items (i.e., the items with odd numbers: 1, 3, 5, 7, 9, 11, 13, 15, 17, 19, 21, 23, 25, 27) assess the tendency to avoid asserting power and authority, as well as the tendency to avoid competitions and disputes. The 14 over-activation items (i.e., items with even numbers: 2, 4, 6, 8, 10, 12, 14, 16, 18, 20, 22, 24, 26, 28) assess the heightened need for a sense of power and control over resources and other people, as well as intense concerns about loss of power. The items are rated on a 7-point scale, ranging from ‘strongly disagree’ to ‘strongly agree’, with higher scores indicating greater deactivation and overactivation of the power system. Items 21 (De) and 26 (Hy) are reversed in the scoring. Finally, the Italian version translated and validated on an Italian sample by Salzano et al. (2023) was used. Cronbach α were 0.833 for the PBSS Deactivation subscale, and 0.827 for the Hyperactivation.

The Reflective Functioning Questionnaire (RFQ; Fonagy et al., 2016) is a tool employed to evaluate mentalization abilities by gauging the level of certainty and uncertainty individuals possess in utilizing information about their mental state to comprehend their own and others’ actions. The current investigation embraced the endorsed 7-point Likert scale, encompassing responses ranging from strongly disagree to strongly agree. Among the 6 items on each subscale, two are distinctive while four are common across both subscales. Within the RFQ_C subscale, the degree of certainty regarding mental states is assessed based on the degree of disagreement with statements like ‘People’s thoughts are a mystery to me’. The items are re-evaluated (3, 2, 1, 0, 0, 0, 0 with 3 = strongly disagree) such that strong disagreement indicates hypermentalizing, whereas any level of agreement (or a neutral response) signifies more authentic mentalizing (acknowledging the opacity of mental states). In the RFQ_U subscale, uncertainty regarding mental states is assessed by the degree to which an individual agrees with statements such as ‘Sometimes I do things without really knowing why’, and is re-evaluated (0, 0, 0, 0, 1, 2, 3; with 3 = strongly agree). Elevated scores indicate a perspective characterized by a lack of understanding of mental states, or ‘hypo-mentalizing’, while lower scores indicate recognition of the opacity of mental states, a hallmark of proficient mentalizing. Both subscales are calculated based on the mean of the 6 items. Due to the four shared items, scored in opposing directions, although each subscale ranges from 0 to 3, the total of the two subscales cannot surpass 4. Consequently, there exists a robust negative correlation between the two subscales, in line with their contrasting interpretations of hypo- and hyper-mentalizing (e.g., Cucchi et al., 2018). For this investigation, the Italian version furnished by Fonagy et al. (2016) and accessible at https://www.ucl.ac.uk/psychoanalysis/research/reflective-functioning-questionnaire-rfq was utilized. Cronbach’s alpha coefficient of 0.77 for RFQ_U and 0.75 for RFQ_C (Morandotti et al., 2018). The structure of the questionnaire has been described in Table 2. This is to provide clarity on how the items are divided into the two subscales and not for psychometric reasons (see Morandotti et al., 2018).

**Table 2:** Items and factors of the Reflective Functioning Questionnaire – 8.

***n***	**Item**	**Factor certainty**	**Factor uncertainty**
1	People’s thoughts are a mystery to me	*RFQ_C*	
2	I do not always know why I do what I do	*RFQ_C*	*RFQ_U*
3	When I get angry, I say things without really knowing why I am saying them	*RFQ_C*	
4	When I get angry, I say things that I later regret	*RFQ_C*	*RFQ_U*
5	If I feel insecure, I can behave in ways that put others’ backs up	*RFQ_C*	*RFQ_U*
6	Sometimes I do things without really knowing why	*RFQ_C*	*RFQ_U*
7	I always know what I feel		*RFQ_U*
8	Strong feelings often cloud my thinking		*RFQ_U*

The short version of the Scale for Interpersonal Behavior (SIB; Arrindell et al., 1984, 2002; Arrindell et al., 2004) is a 25-item multidimensional measure that assesses difficulty and discomfort in asserting oneself across four domains (negative assertion, personal limits, assertiveness initiation and positive assertion). Each domain is assessed in two ways: the probability of response (frequency) and the degree of discomfort (distress) associated with self-assertion attempts. Items are rated on two separate 5-point scales, one for discomfort (from ‘not at all’ to ‘extremely’) and the other for the likelihood of engaging in a specific behavior (from ‘I never do’ to ‘I always do’). In this case, a general assertiveness score was used, specifically the social discomfort scale (SIB_Discomfort). Cronbach αs were 0.90 for the distress score. Raw scores were transformed into standard T-scores as reported by Arrindell et al. (2004).

The Picture Stories Task (PST) is a theory of mind test developed by Brüne (2003) that proposes to measure theory of mind through 6 stories told in 4 pictures each. Each story is represented in 4 pictures that, when ordered in a certain way, make logical sense. The cards are arranged randomly in front of the participant who must rearrange them according to his or her own reasoning. If the sequence is correct, a certain score is awarded depending on the position of each picture. After doing this, specific questions are asked of the participant about the story they have just ordered. The questions concern the reality of the facts, first- and second-order beliefs, first-, second- and third-order false beliefs, detection of deception and cheating. Each question is given a score of 0 if the answer is incorrect, 1 if it is correct. Regarding the order of the stories, a score of 2 is given if the first and last pictures are in the right position and a score of 1 for the middle pictures. The total score for each story is calculated by adding up the score obtained from the order of the pictures and the answers to the questions for each story. The total test score is 59 (PST_tot) and total score Cronbach’s *α* = 0.80.

### 2.3 Procedures

Each participant was individually tested in our laboratory at the Disability Research Centre (DRC) (Department of International Humanities and Social Sciences, Rome University of International Studies) in a single session lasting about 45 min. After filling out an informed consent form, participants also completed a personal data form, including sex, age, native language, and anamnestic data on past and current psychiatric, neurological, and neurodevelopmental conditions. Then, participants completed the 28-item Italian version of the PBSS, the Reflective Functioning Questionnaire (8-items version), the short version of Interpersonal Behavior Scale (SIB), and the Picture Stories Task (following Brüne, 2003 for the administration). The Power Behavioral System Scale (PBSS) is a comprehensive self-report measure designed to evaluate an individual’s overall approach to power and assertion. Developed by Shaver et al. (2011), the PBSS consists of 28 items that are divided into two primary categories, each reflecting different strategies for managing power dynamics: deactivation and hyperactivation. The deactivation items, found in the odd-numbered questions (1, 3, 5, 7, 9, 11, 13, 15, 17, 19, 21, 23, 25, 27), measure a person’s tendency to shy away from asserting power and authority. This includes an inclination to avoid competition and conflict. Higher scores on these items suggest a greater tendency to deactivate power and disengage from power-related situations. Item 21 is reverse scored to account for any inherent biases in the responses. The hyperactivation items, found in the even-numbered questions (2, 4, 6, 8, 10, 12, 14, 16, 18, 20, 22, 24, 26, 28), assess an individual’s heightened need for power and control over resources and people, along with intense concerns about losing power. High scores in this category indicate a strong hyperactivation of the power system. Item 26 is reverse scored to ensure accurate measurement. Participants rate each item on a 7-point Likert scale, ranging from ‘strongly disagree’ to ‘strongly agree.’ Higher scores on the deactivation items indicate a greater tendency to avoid power, while higher scores on the hyperactivation items reflect a greater need for power and control. The PBSS has demonstrated strong internal consistency, with Cronbach’s alpha values of 0.85 for the hyperactivation items and 0.90 for the deactivation items, indicating high reliability in measuring these constructs (Salzano et al., 2023). The Theory of Mind Picture Stories Task (ToM PST, ‘Cartoon Test’) (Brüne, 2003) contains both lower-order and higher-order ToM tasks. It evaluates mind-reading abilities through actions performed by cartoon characters and includes questions regarding first-, second-, and third-order ToM. Additionally, it contains questions measuring comprehension of reciprocity, deception, and cheating. It employs not only pictorial tasks but also assesses verbal ToM skills and controls potential interference with attention impairments through questions monitoring comprehension (Fekete et al., 2022). The Reflective Functioning Questionnaire (RFQ) was developed as a brief, easy-to-administer screening measure of reflective functioning (Fonagy et al., 2016). The Scale for Interpersonal Behavior (SIB) is a 50-item multidimensional measure of difficulty and distress in assertiveness. The SIB assesses negative assertion, expression of and dealing with personal limitations, initiating assertiveness, and positive assertion (Nota et al., 2011).

### 2.4 Statistical analysis

First, we perform an independent samples t-test to compare levels of hyperactivation and deactivation of the power behavioral system (PBSS-Hy; PBSS-De) based on the participants’ gender. To verify the assumptions of normality and equal variance, Shapiro–Wilk (PBSS-Hy: W = 0.980, *p* = 0.360; PBSS-De: W = 0.983, *p* = 0.501) and Levene tests [PBSS-Hy: *F*(1,65) = 0.639, *p* = 0.427; PBSS-De: *F*(1,65) = 2.978, *p* = 0.089] were performed for gender.

Second, we ran a simple linear regression analysis to determine how the quantitative variables hyperactivation (PBSS - Hy) and deactivation (PBSS - De) could be predicted by performance on the ToM test, mentalization and assertiveness, in terms of social distress. For the simple regression analysis of hyperactivation level, we checked for the assumptions of multicollinearity (variance inflation factor or VIF higher than 5 or 10 was a cause of concern; none of the variables presented a VIF higher than 5 or 10; James et al., 2013). Moreover, to verify the assumptions of normality and autocorrelation, Shapiro–Wilk (PBSS-Hy: *W* = 0.979, *p* = 0.316; PBSS-De: *W* = 0.982, *p* = 0.444) and Durbin-Watson tests [PBSS-Hy: DW = 1.813, *p* = 0.480; PBSS-De: DW = 2.418, *p* = 0.108] were performed. All the statistical analyses were performed using the open access software Jamovi version 2.3 (R Core Team, 2021; The jamovi project, 2022) and a *p*-value <0.05 was considered statistically significant.

## 3 Results

To test whether there were gender differences in PBSS hyperactivation and deactivation strategies, a two-tailed independent samples t-test was performed by entering Gender as a fixed factor and the two PBSS dimensions (Hy and De) as quantitative dependent variables. The analysis showed no significant differences in the two PBSS dimensions based on gender [PBSS – Hy: *t*(65) = 0.946, *p* = 0.348, 95% CI: −3.116, 8.727, *d* = 0.236; PBSS – De: *t*(65) = 0.096, *p* = 0.924, 95% CI: −5.419, 5.965, *d* = 0.024] (Table 3).

**Table 3:** Means and standard deviations of hyperactivation and deactivation levels in males and females.

	**Mean**	**SD**	***p***
PBSS – Hy – Females	47.25	11.71	
PBSS – Hy – Males	44.44	12.17	0.348
PBSS – De – Females	46.12	12.47	
PBSS – De – Males	45.85	9.68	0.924

**Figure 1:**
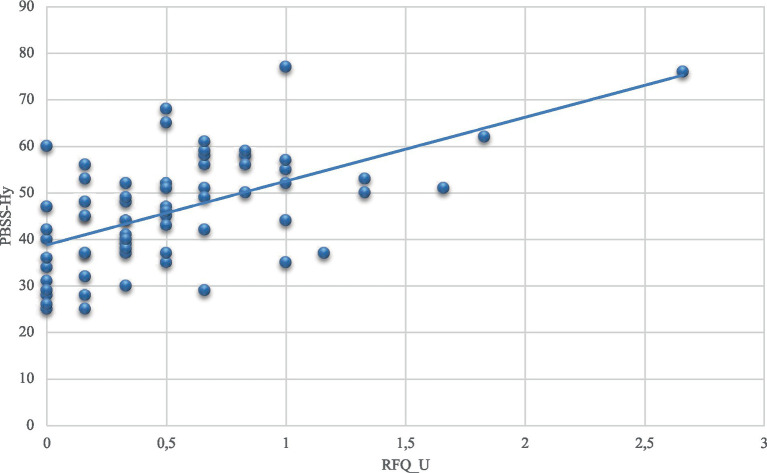
Scatter plot of the PBSS - Hy and RFQ_U.

A simple linear regression analysis was conducted to determine how well the variables hyperactivation and deactivation could be predicted by five quantitative variables: Age, PST_total, RFQ_C, RFQ_U, and SIB_Discomfort. For the hyperactivation variable, the five independent variables combined explained 29.8% of the variance of the PBSS-Hy variable, representing a significant proportion of explained variance, adjusted *R*^2^ = 0.298, *F* (5,61) = 6.593, *p* < 0.001. It is also evident that the variable “RFQ_U” significantly predicts hyperactivation levels (*t* = 3.622, *p* < 0.001; Figure 1): by analyzing the β coefficients of the model, it can be predicted that for each additional point on the hyperactivation scale, the score on the RFQ_U scale increases by 11.387 points. The variables “Age,” "RFQ_C,” “SIB_Discomfort,” and “PST_total” were not significant predictors of hyperactivation levels (*t* = −1.069, *p* = 0.289; *t* = −0.838, *p* = 0.405; *t* = 0.839, *p* = 0.404; *t* = 0.208, *p* = 0.836): for each additional point on the hyperactivation scale, age decreases by −0.536 years, the score on the RFQ_C scale decreases by –1.829 points, the score on the SIB_Discomfort scale increases by 0.074 points, and the PST_total score increases by 0.130 points. A block model selection procedure was used to select the best predictors, starting with the variable Age and then gradually adding the remaining predictors (RFQ_U, RFQ_C, SIB_Discomfort and PST_total) and comparing the models statistically. The best model includes the predictors ‘Age’ and ‘RFQ_U’ [adjusted *R*^2^ = 0.312, *F*(2,64) = 15.996, *p* < 0.001; Table 4].

**Table 4:** Regression analysis of hyperactivation levels with RFQ_C and RFQ_U as predictors.

**Predictor**	***β***	**Standard error**	**95% CI LL**	**95% CI UL**	***t***	***p***
Intercept	51.496	12.444	26.636	76.356	4.138	**<0.001**
Age	−0.484	0.471	−1.425	0.456	−1.029	0.308
RFQ_U	13.314	2.496	8.327	18.300	5.334	**<0.001**

Statistical significances are in bold.

Considering the deactivation variable, only three independent variables (RFQ_C, RFQ_U, and age) were considered due to the assumption of multicollinearity. This combination of variables explained 5.8% of the variance of the PBSS-De variable, which does not represent a significant proportion of explained variance, adjusted *R*^2^ = 0.058, *F*(3,63) = 2.350, *p* = 0.081. The variables “RFQ_C,” “RFQ_U,” and “Age” did not significantly predict deactivation levels (*t* = −1.745, *p* = 0.086; *t* = 0.305, *p* = 0.761; *t* = −0.914, *p* = 0. 364): specifically, for each additional point on the deactivation scale, the RFQ_C scale score decreases by −3.753 points, the RFQ_U scale score increases by 1.059 points, and age decreases by −0.482 years (Table 5).

**Table 5:** Regression analysis of deactivation levels with RFQ_C, RFQ_U and age as predictors.

**Predictor**	***β***	**Standard error**	**95% CI LL**	**95% CI UL**	***t***	***p***
Intercept	62.768	14.648	33.497	92.038	4.285	**<0.001**
RFQ_C	−3.753	2.150	−8.049	0.544	−1.745	0.086
RFQ_U	1.059	3.470	−5.875	7.993	0.305	0.761
Age	−0.482	0.527	−1.535	0.571	−0.914	0.364

Statistical significances are in bold.

## 4 Discussion

The objective of this study is to assess the levels of hyperactivation and deactivation within the motivational system of dominance using a self-report questionnaire. Initially, we examined potential gender disparities in hyperactivation and deactivation levels within our sample. Contrary to our expectations, findings indicated no difference in variance in dominance or submission behavior perception based on gender. Consistent with Tang-Smith et al. (2015), gender was not predictive of any dominance behavioral system component, suggesting involvement of other variables aside from gender. A potential interpretation of this result may pertain to the manner in which dominance is assessed in our study. This variable may not be associated with biological gender but rather with other external factors (e.g., environmental, social, etc.). Additionally, it should be considered that our sample consists of young, educated individuals who might express their dominance differently than other groups within the population.

Dominant and submissive behaviors are widely considered evolutionarily advantageous, aiding in conflict regulation and resource acquisition, typically favoring reproductive success (Fournier et al., 2002). Various behavioral strategies for acquiring power have been identified, categorized along a spectrum of prosocial to aggressive approaches (Zuroff et al., 2010). Sex-specific disparities in neural mechanisms governing social and aggressive behavior expression are probable due to distinct evolutionary pressures on males and females (Terranova et al., 2016). In males, the Dominance System often manifests as coercive social dominance, contrasting with females’ inclination toward prosocial dominance strategies (Hawley et al., 2002). The onset of sexual maturity marks the Dominance/Submission system’s activation, primarily driven by gonadal testosterone production, notably higher in males compared to females, whose hormonal system is prominently influenced by estrogens and menstrual dynamics (Giacolini et al., 2021). Despite these biological distinctions, no gender disparities were observed in the hyperactivation and deactivation variables. It’s pertinent to note that our sample comprises a non-clinical population, whereas extreme cases or clinical presentations of dominance behaviors often involve aggression, whether physical or verbal, directed externally or internally (Gibson and Galea, 2023). In social species, aggression significantly contributes to establishing dominance hierarchies essential for group stability, granting dominant individuals’ advantages such as power, access to resources, and mating opportunities. Consequently, dominance and competitive aggression represent fundamental social strategies crucial for fostering and maintaining relationships in social species (Gibson and Galea, 2023). Although aggression and dominance have biological and ethological foundations, problematic violent outbursts signify pathological conditions (Miczek et al., 2007). In humans, aggression and violence are prevalent among adults with borderline and antisocial personality disorders, schizophrenia, posttraumatic stress disorder (PTSD), bipolar disorder, substance abuse, attention-deficit/hyperactivity disorder (ADHD), and various neuropsychiatric conditions, including dementia (Rosell and Siever, 2015). Intriguingly, gender differences are observed in the incidence and clinical progression of many of these psychiatric disorders, with males disproportionately affected. Moreover, these differences extend to the phenotypic expression of aggression, with males exhibiting more physical aggression while females tend toward indirect aggression (Wranghama, 2017), such as spreading rumors, social exclusion, and criticism of potential rivals (Vaillancourt, 2013). We interpret our findings as being due to the fact that we consider a non-clinical population. Maybe gender differences in activation of the dominance behavioral system might emerge in the presence of clinical conditions. However, it is necessary to investigate other possible variables that may influence levels of activation of the dominance motivational system in a non-clinical population (such as level of testosterone, mood, socio-economic level). Furthermore, in the regression analysis, age did not significantly predict hyperactivation or deactivation levels. This might indicate that chronological age alone does not strongly influence these dimensions of dominance in the studied population. Other developmental factors or life experiences not captured by age alone could play a more significant role in how individuals express dominance-related behaviors.

Additionally, our findings reveal that hyperactivation levels within the dominance behavioral system were predicted by mentalization abilities. Specifically, increased hyperactivation correlated with heightened hypomentalization. We found a discrepancy in levels of activation of the dominance system (hyperactivation and deactivation) related to mentalization skills (hypo and hyper-mentalization). The levels of explained variance are higher at the levels of hyperactivation, suggesting that factors influence hyperactivation more than deactivation. The regression model used seems to be able to explain the variance in the data more for hyperactivation levels than the variance in the data for deactivation levels. It is possible that the constructs measured in the study (such as reflective functioning, social discomfort, etc.) have differential impacts on these aspects of dominance behavioral systems. Specifically, the construct of mentalization does not explain the deactivation of the dominance system, probably because deactivation of the system presupposes a good ability to mentalize and infer others’ mental states without over-mentalizing (hypermentalization) or being ambiguous (hypomentalization). Mentalization is the process of understanding subjective states and mental processes. It is crucial for fostering robust self-awareness, constructive social interactions, and relational mutuality (Bateman and Fonagy, 2010). Hypomentalization, characterized by an inability to consider complex mental models, is associated with various psychopathologies (Bateman and Fonagy, 2004, 2008; Skårderud, 2007a,b; Luyten et al., 2012), often contributing to psychological distress in social interactions (Steinmair et al., 2021). Steinmair et al. (2021) observed significant improvements in mentalization skills following mentalization-based therapy (MBT), leading to enhanced mental health outcomes. Notably, high levels of hypomentalization are typical in dysfunctional personalities such as borderline personality disorder (Fonagy et al., 2016), characterized by emotion regulation difficulties, impulse control issues, and interpersonal instability (Posner et al., 2002). Conversely, individuals with proficient mentalization skills typically exhibit resilience in stressful conditions, fostering positive perspectives despite adversity (Fonagy et al., 1994). They demonstrate adeptness in forming supportive relationships and effectively managing stress (Hauser et al., 2006; Luyten et al., 2009), often displaying creativity, symbolization abilities, and a tendency for exploring internal and external worlds (Bateman and Fonagy, 2013). Moreover, research across animal, biological, and behavioral domains suggests a robust association between dominance system dysfunctions and various psychiatric conditions including psychopathy, antisocial personality disorder, alcohol-related issues, depression, anxiety disorders, and bipolar disorder (Trower and Gilbert, 1989; Gilbert et al., 2007; Johnson et al., 2012), wherein compromised mentalization is also implicated. Considering these perspectives, mentalization appears intricately linked with the dominance behavioral system, given its focus on dominance and power dynamics in interpersonal interactions. Despite our non-clinical sample, hyperactivation levels were inversely associated with mentalization proficiency, suggesting the importance of mentalization skills in the appropriate activation of the dominance behavioral system. Our findings showed that individuals scoring higher on the hypomentalization scale, which could indicate a lower capacity for reflective functioning (mentalization), tend to exhibit higher levels of hyperactivation. Possible explanations could include difficulties in understanding others’ mental states or emotions, which might lead to more dominant behaviors as a compensatory mechanism. According to Liotti’s perspective (Liotti, 2001a,b), this aspect is important for the interpersonal relationships which are always regulated and motivated by these motivational systems, including DBS. In a non-clinical population, it is worth emphasizing what cognitive processes come into play within interpersonal relationships. Through our results, we can claim that lower capacities of mentalization lead to an increase in activation of the dominance system, creating conditions of conflict between people in terms of aggressive and dominant behavior. Furthermore, deactivation levels within the dominance system were not predicted by mentalization or age. Our findings do not allow us to confirm previous research that highlights the correlation between deactivation and assertiveness deficits (Shaver et al., 2011). Other studies also support the association between submissiveness and social discomfort (Salzano et al., 2023; Zappullo et al., 2023).

Lastly, our study found no significant relationship between Theory of Mind test performance and hyperactivation or deactivation levels. The RFQ and PST measure aspects of reflective functioning and theory of mind, respectively, but they may emphasize different dimensions or nuances. The finding that RFQ-U predicted hyperactivation while PST did not could be attributed to the nature of the test: RFQ might capture nuances related to uncertainty or ambiguity in mentalizing processes that are more relevant to hyperactivation behaviors, while PST assesses first- and second-order beliefs, first-, second- and third-order false beliefs, detection of deception and cheating. They are different aspects than the construct of mentalization. In addition, we attribute this to the test’s design by Brüne (2003) for pathological populations, rendering it less suitable for identifying differences in non-clinical populations. Finally, these results may have significant implications for improving techniques in psychotherapies: (i) understanding that high levels of hyperactivation are predicted by hypomentalization can help therapists tailor treatment for patients. For example, they might focus on specific interventions to increase awareness and understanding of one’s own and others’ emotions and thoughts (Bateman and Fonagy, 2013); (ii) therapists may use this information to educate patients about the links between mentalization and hyperactivation of DBS. This can help patients better understand their emotional and behavioral reactions and adopt strategies to manage them more effectively; (iii) developing of specific interventions to enhance patients’ mentalization skills. For instance, mindfulness techniques or reflection exercises could be introduced to help patients become more aware of their mental states and those of others. This is important in Cognitive Behavioral Therapy (CBT), which is currently focused on the use of techniques such as mindfulness (Apolinário-Hagen et al., 2020).

## 5 Conclusion

In conclusion, this study suggests the potential existence of a correlation between mentalization, assertiveness, and dimensions of dominance/submission. Specifically, mentalization appears to predict hyperactivation (dominance), while it does not predict deactivation (submission). Such relationships could prove beneficial in treating various psychopathologies, by understanding their clinical characteristics in terms of these dimensions. To achieve this, there is a significant need for studies comparing multiple psychopathologies using self-report, observational, and biological measures of the dominance behavioral system, while accounting for factors such as age, gender, social context, as well as symptom profiles and severity. Moreover, our study has some limitations that warrant consideration: (a) the sample size precludes generalization of results; (b) other variables that may influence the reported relationships were not considered. As a prospective avenue for further research, we recommend conducting comparisons between groups with psychopathologies to understand how dimensions of the dominance behavioral system manifest within specific pathologies and how they correlate with the variables outlined in this study. This may assist therapists in identifying patients’ most significant challenges.

## Data availability statement

The raw data supporting the conclusions of this article will be made available by the authors, without undue reservation.

## Ethics statement

The studies involving humans were approved by Comitato Etico per la Ricerca Umanistica e Sociale (CERUS). The studies were conducted in accordance with the local legislation and institutional requirements. The participants provided their written informed consent to participate in this study.

## Author contributions

FC: Conceptualization, Data curation, Formal analysis, Investigation, Methodology, Project administration, Writing – original draft. CE: Conceptualization, Data curation, Formal analysis, Investigation, Methodology, Project administration, Writing – original draft. IP: Writing – review & editing. LSS: Writing – review & editing. AF: Resources, Supervision, Validation, Visualization, Writing – review & editing.
